# Medical and Philosophical News

**Published:** 1784

**Authors:** 


### SECTION III.

### Medical and Philosophical News.

THE Royal Society at Montpellier have given notice, that one of their members (M. Broussonet, of Paris, F. R. S.) has remitted to them the sum of 300 livres, as a premium for the best Eulogium of Peter Richer de Belleval, the first Professor of Anatomy and Botany in the University of Montpellier.

pellier. This Profeſſor, to whom the Univerſity is indebted for the eſtabliſhment of its botanic garden in 1598, expended his whole fortune in the cultivation of botany. A conſiderable number of plates, intended for a very extenſive botanic work, which he did not live to complete, are ſtill extant, and are ſaid to be engraved with a degree of accuracy unequalled before his time. The pieces on this ſubject muſt be written in Latin or French, and ſent to M. de Ratte, Secretary of the Society, before the 30th of Sept. 1785.

---

The Royal Medical Society at Paris have propoſed the following prize queſtion, for a prize of 600 livres: "What are the characte-"riſtic ſymptoms of nervous diſorders, pro-"perly ſo called, ſuch as hyſteria, hypochon-"driaſis, &c.; how far do they differ from other "analogous diſeaſes, ſuch as melancholia, for "example; what are their principal cauſes, "and the general indications to be obſerved in "their treatment?" Diſſertations on this ſubject muſt be ſent to M. Vicq D'Azyr, ſecretary of the Society, before the 1ſt of January, 1786.

---

M. Geoffroy

M. Geoffroy formerly procured five ounces of water from eight ounces of spirit of wine; but M. Lavoisier has lately, by combustion, not only converted the whole of the spirit of wine he employed in his experiment into water, but has done even more, having from 16 oz. of spirit procured 18 oz. of pure water. The addition was the result of the decomposition of the air employed in the combustion of the spirit of wine.

---

The *Globularia Alipum* of Linnæus has lately been recommended as a purgative remedy, by M. Ramel, junior, Physician at Aubagne, in Provence, where it is said to be in common use. An ounce of the leaves of this shrub, which grows in many parts of Provence, are directed to be boiled for this purpose a quarter of an hour in a few ounces of water. This decoction is spoken of as an excellent bitter purge. In smaller doses, it is given with success in intermittents.

---

In the *Journal de Paris* for Oct. 17, M. Alphonso le Roy has published a farther account of of the caſe of the ſection of the ſymphyſis pubis, mentioned in a former part of this volume (*page* 312). From this ſubſequent account we learn, that the patient, who is ſaid to be perfectly recovered, was able on the eleventh day after the operation to get out of bed, and on the thirtieth to walk through the ſtreets of Paris.

---

The Abbé Fontana, in a letter to M. Gibelin, inſerted in the Abbé Rozier's *Journal de Phyſique* for June 1784, gives an account of ſome farther experiments on the poiſon of the viper, which may ſerve as a ſupplement to his late work on that ſubject, (*ſee page* 1). Theſe experiments, which were made in conſequence of the accounts publiſhed in ſome of the Italian Journals, of cures performed by injecting ſpirit of hartſhorn into the veins, prove that no dependence can be placed on this method of treatment.

In the ſame letter alſo the learned Abbé gives ſome additions to his obſervations on the nerves. He has examined, with a lens of great magnifying powers, the gelatinous matter he had formerly mentioned as filling the nervous cylinders, (*ſee page* 26), and he finds it to be

 compoſed composed of minute grains, four or five times less in diameter than the red globules of the blood. This elastic graniform substance he has observed in the nerves of different animals; and if these minute grains may be supposed to constitute the animal spirits, he thinks we may explain the phenomena of animal motion, by supposing this elastic substance to be continued throughout the whole length of the nervous cylinders, and to have an insensible vibration similar to that of the air in the propagation of sounds.

---

To Dr. SIMMONS.

Sir,

A letter I have lately received from a friend on the Continent, containing much useful and curious information, seems to me so exactly to coincide with the intention of that part of the London Medical Journal, comprised under the head of *Medical News*, that I have taken the liberty of sending to you an extract of it, even without waiting the permission of the writer.— If it appears to you in the same light that it does to me, you will probably willingly give it a place a place in the next Number. It comes from an Engliſh Phyſician eſtabliſhed in Italy, a gentleman of great erudition, ſound judgement, and unimpeached integrity. Theſe circumſtances I have thought fit to premiſe, as having ſuppreſſed his name, and as they may tend to impreſs a due degree of confidence on what he has advanced.

I am, Sir, very reſpectfully,

Your obedient, humble ſervant,

*Liverpool,* THOMAS HOULSTON.

*Nov.* 15, 1784.

———" I thank you for your *Obſervations on Poiſons*, and am well pleaſed with your ſucceſs in caſes of dyſentery, depending on old liver-caſes, in treating which I certainly will follow your example the firſt opportunity. The action of poiſons, if well underſtood, might throw much light on practical phyſic; but the various effects produced by them in various perſons, mix with our knowledge much confuſion. One at Paris ſwallowed an ounce, or more, of aqua-fortis; the conſequences were, a ſuſpenſion of ſtools and urine many days, without pain; then a train of painful ſymptoms, which, however, declining ſenſibly for near ſix

 weeks, weeks, gave hope of recovery, when the friend from whom I have the account loſt ſight of him. Another here ſwallowed leſs, I believe, than a drachm of aqua-fortis, and though ſoap and milk were pretty quickly adminiſtered, died in about forty hours.——A perſon here chewed and ſwallowed five cantharides, and went about his uſual buſineſs. Two or three hours afterwards, feeling a heat in his ſtomach, he applied to me to know if any miſchief would enſue. I had him bled, and filling his ſtomach with oil, made him vomit, which he did pretty copiouſly, bringing up conſiderable quantities of membranes, which appeared to be the beſt part of the lining of the œſophagus and ſtomach. Half of his tongue was ſtripped. I ordered him to drink milk copiouſly; though I confeſs now, without much hope; and a ſtrangury coming on, I rubbed the pubis and perinæum with camphor, and covered all with warm fomentations. The ſtrangury went compleatly off in leſs than two hours, and next morning he had an erection, which was not troubleſome, nor continued above half an hour. He felt a ſoreneſs in the ſtomach for two or three days, but no farther inconvenience.—How different this, from the uſual hiſtories of thoſe who have taken cantharides even in a much leſs doſe!

"We have lately ſeen here one, who, in the hydrophobia canina, had an interval of ſixteen or more hours, in which he drank, eaſily and without agitation, large quantities of various liquors.—Of nine perſons in the ſame priſon, bit by the ſame dog, one only was attacked with the hydrophobia; and he neither the firſt nor the laſt that was bit, nor the moſt wounded. He fell ſick more than four months after the bite, was under my care, and died hydrophobous and convulſed, but without delirium.—Not long ago a gentleman died here, who had reſiſted incredible quantities of arſenic. It ſeems he had taken, in eight or ten times, about one third of an ounce, without its producing the deſired effect, or even bringing on ſuch ſymptoms as to raiſe ſuſpicion of poiſon. At laſt, a whole ounce was put into a ſaucepan of broth, of which he drank a good cupfull. I think he did not ſwallow then, at once, ſo little as half a drachm of arſenic, yet he ſurvived four or five days. I don't, however, infer, from the irregularity and incertitude of the action of poiſons, that we ought not, or cannot, counteract them. Perhaps even every poiſon may have have its ſpecific antidote, as ſimple as the poiſon itſelf, but till we know theſe, we muſt content ourſelves with making the beſt uſe we can of the means we are poſſeſſed of, though generally imperfect. Theſe you have well diſplayed in your account, &c. where you have very juſtly obſerved, that frequently life depends on a very quick evacuation of the poiſonous ſubſtance, one of the firſt effects of which is to render the ſtomach paralytic. If people did but think of it, it would ſeldom be neceſſary to loſe time in ſending to an apothecary for an active vomit; a pinch of ſnuff, or a little powdered tobacco, would do the buſineſs.

"We are here attacking cancers with the raw fleſh of the common ſmall lizard, in the doſe of two or three in a day, (*ſee p.* 97). Our neighbours boaſt of ſucceſs in the true cancer. I think I have found great advantage from it in a terrible cutaneous caſe, in which the face, and part of the head, were covered with confluent ulcerated warts; yet I will not chime in with thoſe who extol it, till I am ſurer of its real efficacy.

"People in England ſeem to be, in one point, under a miſtake, with regard to the red Peruvian bark. This is not the ſort eſteemed either in in Spain or in Peru; at leaſt it is not what is ſent thence, by the Governor, for the uſe of the King and royal family, nor what comes in preſents to the great people. I have had ſeveral ſamples of what is ſent to the Duke Grimaldi, and have now before me ſome ounces of what was ſent by the King of Spain for the uſe of the Duke and Ducheſs of Parma. Theſe ſamples are in quills, about the ſize of my little finger, and ſuch as I remember moſt eſteemed in England. We have here the chief, and ſecond apothecary of the late Jeſuits in Alcala, who both tell me, that ſuch is the bark moſt eſteemed by thoſe Fathers, and by the Spaniards in general. The red bark certainly comes in cheſts promiſcuouſly with the reſt, and thoſe cheſts have hitherto been ſold cheapeſt, which had the greateſt proportion of red bark. Not more than eighteen months ago, when the quill bark ſeparately ſold here for three ſhillings and ſixpence, ſterling, the pound, the red bark was ſold at fourteen pence. I am, however, convinced, from my own experience, that the red bark beſt ſtops agues. There are ſometimes mixed with the bark, pieces of a knotty root, called *calaguela*, which is ſaid to have performed lately, at Rome, wonders in dropſical caſes. I

have have ſent to Cadiz for ſome of it, and will endeavour to give it a fair trial.

"You know, that in preparing ſweet ſublimate of mercury, a red powder is found, in part, ſublimed at the bottom of the cake, and in part looſe in the bottom of the ſubliming veſſel. This powder has been taken for crocus of iron, but is in fact ſilver, as you may eaſily prove, by reducing it with the black flux. I don't wonder how the ſilver comes there, but I cannot account for its ſubliming in that form and manner.

"You muſt not expect medical news of much conſequence from Italy in theſe days.—If any thing of this kind, worth reading, is publiſhed, I generally find it is ſome doctrine broached in the Engliſh ſchools, and attempted to be explained in Italian."

---

Laceration of the uterus in parturition is an accident which fortunately does not often occur, as it has hitherto in almoſt every inſtance proved ſpeedily fatal. In one caſe, however, which happened ſome time ago, under the care of Dr. Garthſhore, one of the Phyſicians of the Britiſh Lying-in Hoſpital, the patient lived twentytwenty-fix days after this dreadful accident; and in another and ftill more recent inftance of the fame kind, under the care of Dr. Douglas, one of the Phyficians of the London Lying-in Charity, the patient recovered without any alarming fymptom, and is now alive and in good health. In both thefe cafes, the fœtus, and in the latter of the two the fecundines alfo, which had efcaped into the cavity of the abdomen, were brought back through the laceration, and extracted by the natural paffages.

---

Dr. Hope, Profeffor of Botany at Edinburgh, has lately tranfmitted to the Royal Society a botanical defcription and drawings of the fpecies of *ferula* that produces affafœtida, (*fee* vol. IV. *p.* 421). The figure given of this plant by Kæmpfer, in his *Amoenitat. Exotic.* is now found to be inaccurate.—Dr. Percival, of Manchefter, has communicated to the Society fome obfervations on the acid of tar; the ftrength of which, compared with that of the acid of vitriol, he finds to be as 1 to 14. Dr. Percival thinks it likely to be of ufeful application in

 pharmacy, pharmacy, particularly in the preparation of the ſugar and extract of lead.

